# Harnessing the Tet-Off system for effective gene silencing in *Magnaporthe oryzae*

**DOI:** 10.3389/fpls.2025.1641730

**Published:** 2025-09-12

**Authors:** Zhenyu Fang, Fan Yang, Yongge Ma, Yan Cai, Xin Chen, Shiyu Yang, Abubakar Yakubu Saddeeq, Zonghua Wang, Naweed I. Naqvi, Wenhui Zheng

**Affiliations:** ^1^ State Key Laboratory of Agricultural and Forestry Biosecurity, College of Plant Protection, Fujian Agriculture and Forestry University, Fuzhou, China; ^2^ Key Laboratory of Bio‐pesticide and Chemistry Biology, Ministry of Education, College of Plant Protection, Fujian Agriculture and Forestry University, Fuzhou, China; ^3^ Temasek Life Sciences Laboratory, Department of Biological Sciences, National University of Singapore, Singapore, Singapore; ^4^ Institute of Oceanography, Minjiang University, Fuzhou, Fujian, China

**Keywords:** rice blast fungus, Tet-Off, conditional knockdown, doxycycline, essential genes

## Abstract

Rice blast is the most serious fungal disease affecting rice production worldwide. Studying gene function in the rice-blast fungus *Magnaporthe oryzae* often presents significant challenges, especially if it involves loci that are essential for the fungal growth and/or viability. Researchers expend considerable effort on the knockout process, only to find their attempts end up in vain. To address this, we introduced a transcriptional regulation system based on the Tet-Off system, designed to conditionally and effectively inhibit the expression of target genes using a tetracycline-controlled module in *M. oryzae*. Using *Agrobacterium tumefaciens* mediated-transformation, a Tet-Off cassette was first inserted upstream of the target gene. Stable transformants were obtained in which the gene expression was significantly reduced in the presence of doxycycline. We successfully generated the Tet-Off system to silence two categories of genes—those leading to mild or to severe defects in hyphal growth —demonstrating its effectiveness for functional study of genes that are critical for fungal growth, including essential genes. Additionally, the Tet-Off system, when combined with various fusion tags, enabled effective monitoring of target gene expression and protein localization. This system thus provides a robust and powerful tool for analyzing gene function in fungi without the need for complete gene deletion analysis, and thus is applicable to essential genes in *M. oryzae*, deepening our understanding of their roles in fungal development and pathogenesis.

## Introduction

Rice blast is the most serious fungal disease of rice caused by *Magnaporthe oryzae* (syn. *Pyricularia oryzae*), which poses a major threat to global rice production due to a significant reduction in rice yield (Khush, 2005; Fisher et al., 2012). Usually, the prevention and control of rice blast mainly relies on the use of fungicides. However, there is a lack of effective antimicrobials to prevent and control rice blast, and the long-term and large-scale use of fungicides alone, as well as the high mutation rate and short generation time of the rice blast fungus itself, have led to increasing drug resistance in this pathogen (Tleuova et al., 2020; Li et al., 2020; D’Ávila et al., 2021; Zhang et al., 2024). The search for new molecular targets in the rice blast fungus through research into functional genes is crucial for in-depth understanding of the pathogenicity mechanism and for developing effective fungicides.

Reverse genetics is often used to test the functions of genes in the rice blast fungus (Bhadauria et al., 2009). However, gene knockout often comes with challenges, especially when targeting genes that are critical for the survival of the fungus (Lu et al., 2014). Moreover, obtaining the correct transformants can be labor-intensive and time-consuming, often requiring great efforts with little success, a problem exacerbated by differences in transformation efficiency and the technical complexity involved in the procedures (Zhang et al., 2019; Wang et al., 2024). When targeted gene knockout is unsuccessful, researchers often switch to RNA interference (RNAi) as an alternative approach. However, the silencing efficiency of different RNAi transformants varies greatly, and a large number of transformants need to be screened to determine the right strains with effective gene silencing, and this variability together with off-target effects further complicates the interpretation of the experimental results (He et al., 2018; Mei et al., 2019; He et al., 2020). To overcome these obstacles, some researchers use conditional promoters such as the sodium acetate inducible isocitrate lyase promoter, nitrate reductase promoter etc. to control gene expression (Wang et al., 2003; Kilaru et al., 2015; Zhang et al., 2019). However, these types of promoters come with setbacks as the inducers may affect the mutant’s metabolism, resulting in slow response time and low sensitivity, which may be wrongly interpreted as due to the loss-of-function of the gene. Given these difficulties, more reliable and efficient methods are urgently needed to investigate gene functions, especially in filamentous phytopathogenic fungi.

Doxycycline or Tetracycline-driven transcriptional activation systems are a promising approach to solving these problems. This system is based on the tetracycline resistance operon of the Tn10 transposon of *Escherichia coli* (Berens and Hillen, 2003). Currently, two types of tetracycline regulatory systems, Tet-Off and Tet-On, have been developed. The Tet system consists of two main parts: a promoter/tetracycline transcriptional activator (tTA/rtTA) and a tetracycline-response element (TRE), which consists of a repeated *tetO* sequence and a minimal promoter (Gossen and Bujard, 1992). The Tet system uses tetracycline or its analog doxycycline. Tetracycline is a membrane-permeable antibiotic drug with no molecular targets in eukaryotic. The Tet-Off and Tet-On systems therefore work well in eukaryotic organisms (Gatz, 1997; Wishart et al., 2005). In a Tet-Off system, a tTA that is bound to doxycycline does not bind to *tetO*, and as such, the targeted gene cannot be expressed normally (Takahashi et al., 1986). The Tet-On system is the opposite of the Tet-Off system. In the presence of doxycycline, the trans-transcription activator (rtTA) binds to *tetO* and the gene can be transcribed normally (Gossen et al., 1995; Urlinger et al., 2000). The Tet system plays the role of an effective genetic switch in a variety of organisms including mammals, plants, yeasts and fruit flies, and is the most commonly used chemical regulatory system in mammalian cells (Gossen and Bujard, 1992; Weinmann et al., 1994; Kistner et al., 1996; Das et al., 2016; Yamada et al., 2018; Zhao et al., 2020; McFarland et al., 2020; Jiang et al., 2022). It has also used for the regulation of gene expression in filamentous fungi such as *Aspergillus fumigatus*, *Aspergillus niger*, *Ustilago maydis* and *Phytophthora infestans* (Vogt et al., 2005; Zarnack et al., 2006; Judelson et al., 2007; Meyer et al., 2011; Pinter et al., 2019; van Rhijn et al., 2024).

Our earlier studies have shown the practicality of the Tet-Off system in the rice blast fungus, which not only verified the function of the target gene, but also further investigated the effects of knockdown of the target gene on potential upstream and downstream genes (Yadav et al., 2019; Chen et al., 2023, 2024; Lin et al., 2025). In this article, we present the various control elements of the Tet-Off system, vector construction and genetic transformation strategies, and finally demonstrate the efficacy of our modified system in studying the functions of essential genes in *M. oryzae*. The present study therefore puts forward a simple, fast and effective tool for the control of gene expression, thereby promoting the analysis of gene functions in this model fungal pathosystem. This will essentially accelerate the discovery of potential targets for effective antifungal drugs.

## Materials and methods

### Strains and culture conditions

*M. oryzae* Guy11 was used as the wild-type strain in this study. All strains were cultured in CM medium at 28°C under 12-hour light/dark cycles. For the plate growth assay, colony diameters were measured in triplicate after seven days of incubation, and each growth experiment was repeated three times. For gene expression analysis, mycelial blocks were taken from a 3-day culture on CM medium and transferred to CM liquid culture for 2 days. Dox was then added, and the culture was incubated for an additional 10 h before freezing the mycelia in liquid nitrogen for RNA extraction. Unless otherwise stated, the concentration of Dox used in this study is 30 µM.

### Gene knock-in and complementation

The primers used for amplification and validation of the Tet-Off vector fragments for all genes are listed in **Supplementary Table S2**. The resulting Tet-Off plasmid was sequence-verified and transformed into *A. tumefaciens* AGL1 strain using the freeze-thaw method. Details of all plasmids are provided in **Supplementary Table S3**. ATMT of *M. oryzae* was performed as previously described (Yang and Naqvi, 2014). All complemented strains were obtained through protoplast transformation of the respective Tet-Off strains.

### RNA extraction and RT-qPCR

For gene expression quantification, RNA was isolated using the Eastep Super Total RNA Extraction Kit (Promega, LS1040). The purified RNA was assessed by agarose gel electrophoresis prior to complementary DNA (cDNA) synthesis, which was conducted using the HiScript III RT SuperMix for qPCR (Vazyme, R323). RT-qPCR was carried out on an EASTWIN eQ9600 real-time fluorescent quantitative PCR detection system with Taq Pro Universal SYBR qPCR Master Mix (Vazyme, Q712). The 2^-ΔΔCt^ method was employed to determine the expression levels of target genes (Livak and Schmittgen, 2001). Each sample was tested in three biological replicates. The *M. oryzae* β-tubulin gene (MGG_00604) was used as an internal control, and the primers used are listed in **Supplementary Table S2**.

### Western blotting

Total proteins were isolated from vegetative hyphae using lysis buffer (10 mM Tris-HCl, pH 7.5, 150 mM NaCl, 0.5 mM EDTA, 1% Triton X-100, 1 mM PMSF, and 1× protease inhibitor cocktail) prior to separation by SDS-PAGE. Western blot experiments were performed in triplicate. For Western blot detection of proteins, the following antibodies were used: anti-Actin (Huadingbio, 10011, 1:5000), anti-Flag (Abmart, M20008, 1:5000), anti-Myc (Abcam, ab1326, 1:5000), and Goat Anti-Mouse IgG HRP (Abmart, M21001, 1:5000).

### Confocal microscopy

The mycelia of the involved strains were observed using a Nikon CSU-W1 spinning disk confocal microscope. Detailed parameters are described elsewhere (Chen et al., 2024). The excitation/emission wavelengths used for GFP were 488 nm/500–550 nm.

### Pathogenicity analysis

To evaluate its pathogenicity, conidia were washed from CM culture dishes that had been incubated for 5 days, and the concentration of the spore suspension was adjusted to 3×10^4^/mL, and doxycycline was supplemented into the suspension prior to spray inoculation to sustain target gene suppression. The spray inoculation test was performed on rice CO39 that had been grown for 4 weeks. After 7 days of incubation in a humid environment, the number of lesions was observed and counted. Pathogenicity experiments were performed in triplicate. Unless otherwise specified, all Dox concentrations were set at 30 μM (13.3 μg/mL).

### Accession numbers

Sequence data from this article are available under the following accession numbers: *MoVPS35* (MGG_05089); *MoVMA1* (MGG_08087); *MoTOR* (MGG_15156); pFGL1252_TetOFF (Hyg) (Addgene ID 118992); pFGL1252_TetGFP (Hyg) (Addgene ID 118993).

## Results

### Analyzing the elements, the knock-in process and the working principle of the Tet-Off system

**Figure 1:**
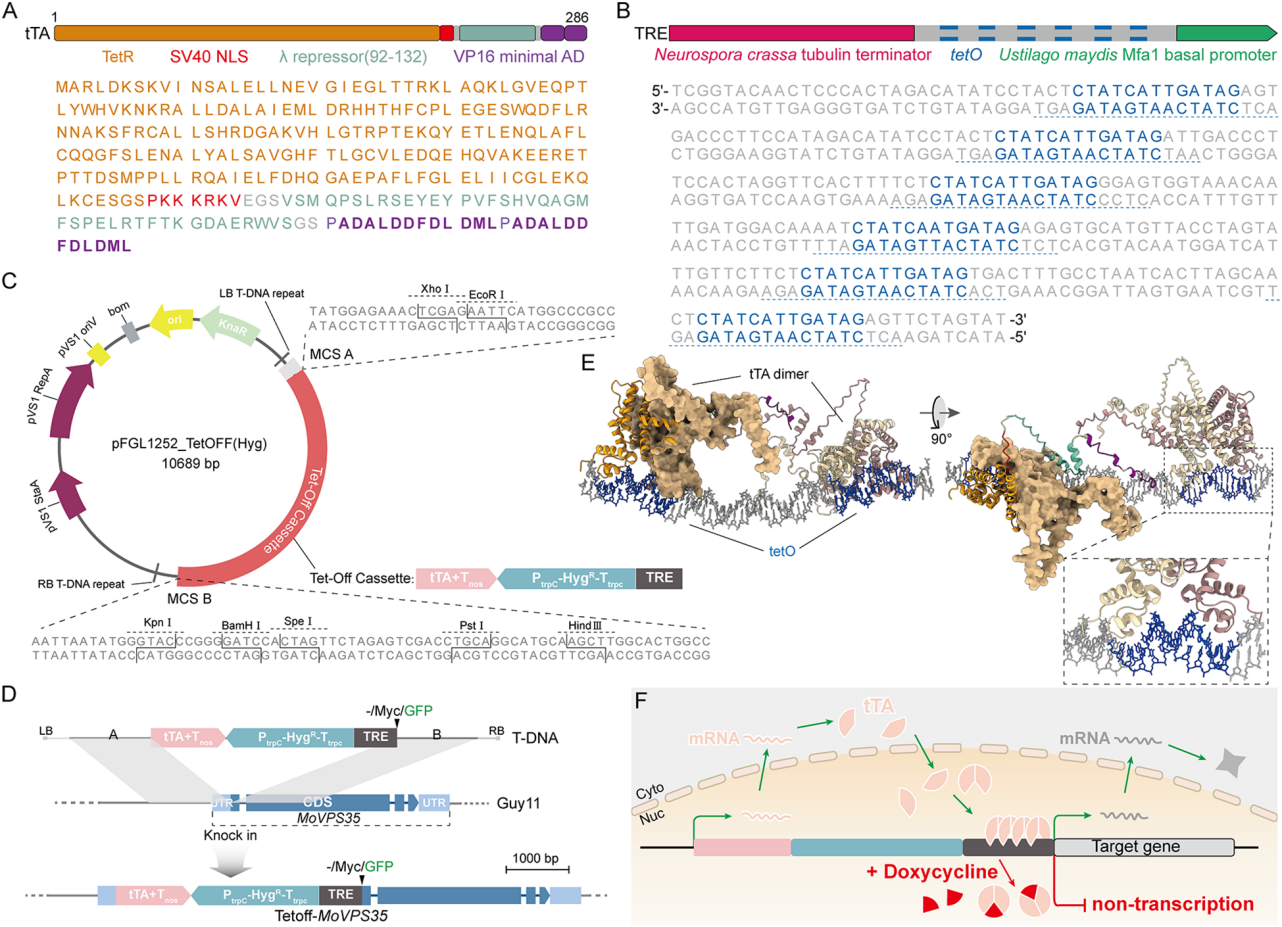
Basic principle of the Tet-Off system in the blast fungus. **(A)** Amino acid sequence and domain composition of tTA. **(B)** Composition of TRE and nucleotide sequence of *tetO* operator elements. **(C)** Map and cloning sites of the ATMT vector pFGL1252_TetOFF (Hyg) for Tet-Off-mediated knock-in. **(D)** Schematic diagram showing the strategy adopted for the insertion of a T-DNA harboring a Tet-Off cassette into *M. oryzae* genome. **(E)** AlphaFold predicts the binding of two tTA dimers into two *tetO* operators. **(F)** Schematic diagram showing the working model of a Tet-Off system in the rice blast fungus.

The Tet-Off system is based on the tTA-*tetO* response element, whereby the tTA protein consists of several key domains. These include a DNA-binding domain of the tetracycline-inducible repressor (tetR), a nuclear localization signal of the large T antigen of SV40 (Simian virus 40), a linker region of the bacteriophage Lambda cI repressor (λ repressor) and two minimal transcriptional activation domains derived from herpes simplex virus protein 16 (VP16) (**Figure 1A**) (Zarnack et al., 2006). The tetracycline-response element (TRE) itself consists of six tandemly arranged *tetO* operators. Downstream of the *tetO_6_* sequence is the basal Mfa1 promoter of *Ustilago maydis*, which drives gene transcription, while upstream is the terminator of the *Neurospora crassa Tubulin* locus, which prevents interference with upstream gene transcription (**Figure 1B**). To facilitate the selection of transformants following Tet-Off transformation, we inserted a hygromycin resistance gene between the tTA and TRE sequences, resulting in a complete 3.8 kb Tet-Off cassette (**Figure 1C**), which was delivered by an *Agrobacterium tumefaciens*-mediated transformation (ATMT) method.

To enable further genetic manipulations, two multiple cloning restriction sites (MCS A and MCS B) were positioned upstream and downstream of the Tet-Off cassette (**Figure 1C**). A fragment (0.7-1.5 kb) upstream of a target gene’s open reading frame (ORF) was cloned into MCS A, while another fragment (0.7-1.5 kb) downstream of the start codon (ATG) of the target gene was cloned into MCS B. This design allows for optional tag insertion at site B for future detection of gene expression (**Figure 1C**). The Tet-Off cassette fused with the target gene fragment was introduced into the *M. oryzae* Guy11 strain via ATMT (Yang and Naqvi, 2014). Following homologous recombination, the Tet-Off cassette was observed to be positioned upstream of the target gene to produce a true Tet-Off transformant (**Figure 1D**). Using this protocol, we obtained effective Tet-Off transformants within two weeks (from vector construction to generation of transformants).

The key mechanism of the Tet-Off system lies in the tTA-*tetO* interaction. Previous studies demonstrated that a tetR protein binds to *tetO* to form a dimer. To further confirm this mechanism, we used AlphaFold to predict the interactions between tTA and *tetO*. The predicted model showed that tTA functions as a dimer, binding to the DNA double helix of the *tetO* operator (**Figure 1E**) (Abramson et al., 2024). In the absence of doxycycline (Dox), tTA is transcribed under the control of the target gene promoter, and upon entering the nucleus, it dimerizes and binds to *tetO_6_* on the TRE. This binding activates the Mfa1 basal promoter, initiating transcription of the downstream target gene. When Dox is added, it binds to tTA, altering its conformation and preventing it from binding to *tetO_6_*. Consequently, the Mfa1 basal promoter is inactivated, halting downstream gene transcription and mRNA production (**Figure 1F**). This dynamic regulatory system allows for precise control of gene expression, making the Tet-Off system a powerful tool for studying essential genes in *M. oryzae*.

### Verifying the effectiveness of the Tet-Off system in *M. oryzae*

To demonstrate the practical application of the Tet-Off system in *M. oryzae*, we selected two distinct target genes for genetic manipulation and transformation. The first gene, *MoVPS35*, plays a minor role in mycelial growth but significantly influences the pathogenicity of *M. oryzae* (Zheng et al., 2015). The other gene, *MoVMA1*, has an ortholog in *A. fumigatus* that was reported to be critical for hyphal growth (Sun et al., 2022). However, *VMA1* orthologs have not been functionally characterized in *M. oryzae* or other plant-pathogenic fungi. Following extensive attempts to delete the *MoVMA1* gene by knockout approach, we were unable to obtain a viable knockout mutant thus far, indicating its crucial role in the survival of the fungus. To test our Tet-Off system, we constructed Tet-Off vectors for *MoVPS35* and *MoVMA1*, respectively, and transformed them into Guy11 wild-type strain and successfully generated positive transformants (**Supplementary Figure S1**). We also generated the respective complementation strains using the target genes in the respective Tet-Off backgrounds.

**Figure 2:**
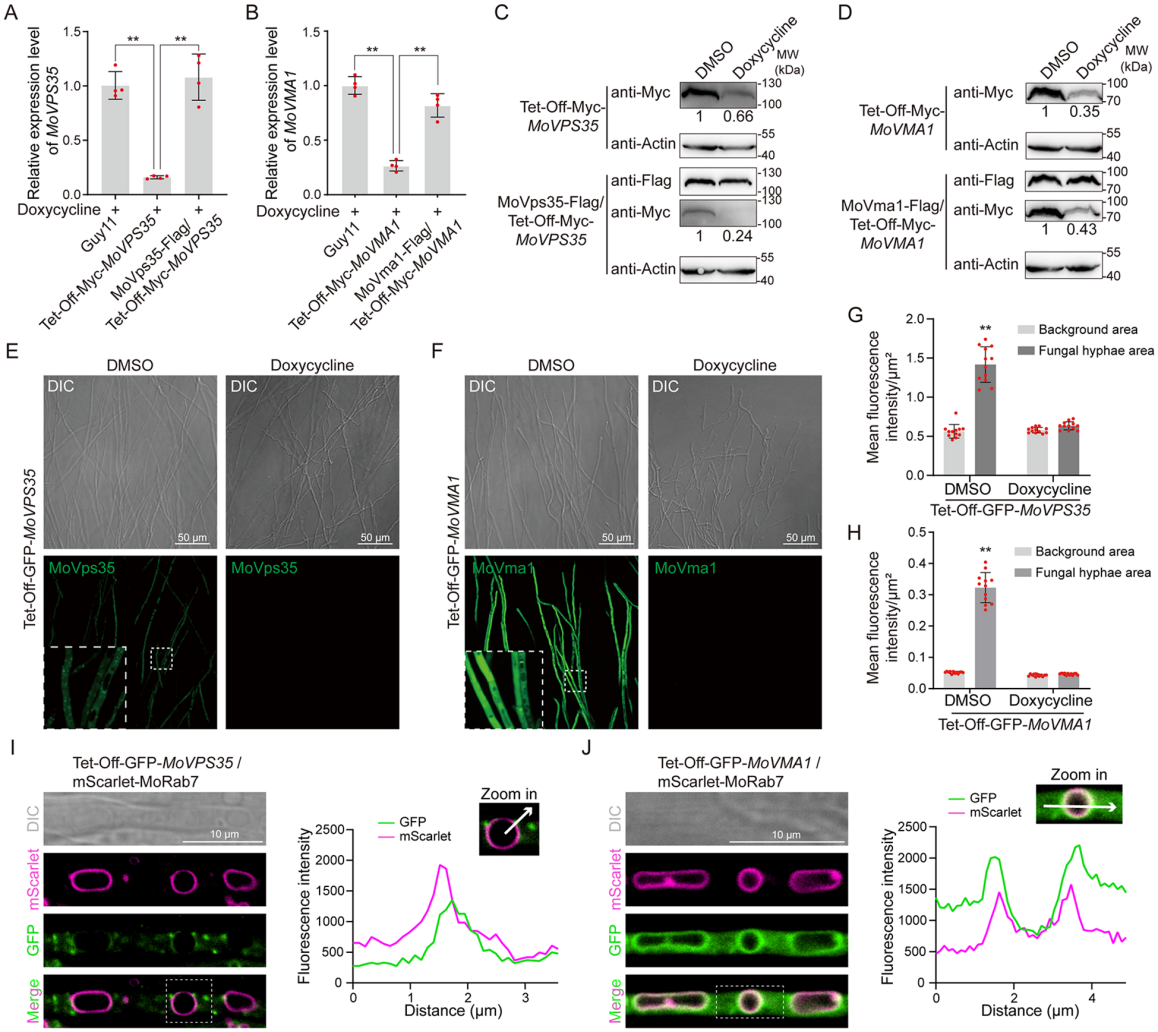
Generation of Tet-Off-mediated knockdown transformants in *M. oryzae*. **(A)** RT-qPCR analysis of the expression levels of *MoVPS35* in *MoVPS35*-Tet-Off mutants (Student’s *t*-test: **, *P* < 0.01). **(B)** RT-qPCR analysis of the expression levels of *MoVMA1* in *MoVMA1*-Tet-Off mutants (Student’s *t*-test: **, *P* < 0.01). **(C)** Analysis of MoVps35 abundance in *MoVPS35*-Tet-Off mutants by immunoblotting (Actin was used as an internal reference). **(D)** Analysis of MoVma1 abundance in *MoVMA1*-Tet-Off mutants by immunoblotting (Actin was used as an internal reference). **(E)** Confocal microscopic examination of GFP fluorescence in GFP-*MoVPS35*-Tet-Off mutants. **(F)** Confocal microscopic examination of GFP signal in GFP-*MoVMA1*-Tet-Off mutants. **(G)** Comparison of GFP fluorescence intensity in GFP-*MoVPS35*-Tet-Off mutants (Student’s *t*-test: **, *P* < 0.01). **(H)** Comparison of GFP fluorescence intensity in GFP-*MoVMA1*-Tet-Off mutants (Student’s *t*-test: **, *P* < 0.01). **(I)** Co-localization of GFP-MoVps35 and mScarlet-MoRab7 in vegetative hyphae. **(J)** Co-localization of GFP-MoVma1 and mScarlet-MoRab7 in vegetative hyphae.

To test the functionality of the Tet-Off system, we measured the relative expression level of *MoVPS35* in Guy11, *MoVPS35*-Tet-Off transformant and the cognate complemented strain, both in the presence and absence of doxycycline (Dox). The results showed that the Tet-Off system significantly reduced the transcription of *MoVPS35* gene, while the gene transcription was effectively restored in the complemented strains (**Figure 2A**; **Supplementary Figure S2A**). Similarly, the relative expression level of *MoVMA1* gene was assessed in Guy11, *MoVMA1*-Tet-Off transformants and the complemented strain, both in the presence and absence of Dox treatment. The results indicated that the Tet-Off system could effectively knock down the *MoVMA1* transcription. This knock down was observed to be restored to normal expression levels in the complemented strains (**Figure 2B**; **Supplementary Figure S2B**). These results demonstrate that the Tet-Off system can precisely and efficiently reduce the transcription of targeted genes in *M. oryzae*.

In addition to measuring the gene transcription levels, we also analyzed the corresponding protein levels under the Tet-Off regulation. Western blot analysis revealed a significant reduction in MoVps35 protein level in the Tet-Off transformants under Dox treatment, while the complemented strain was observed to maintain a normal protein expression level (**Figure 2C**). Similarly, the MoVma1 protein level was significantly down-regulated under Dox treatment in the Tet-Off transformants, with no such effect observed in the complemented strains (**Figure 2D**). These results further support the effectiveness of the Tet-Off system in regulating gene expression in the blast fungus.

To further validate the efficiency and effectiveness of the Tet-Off system in shutting down targeted gene expression in *M. oryzae*, we tagged the genes *MoVPS35* and *MoVMA1* with GFP in the Tet-Off transformants and analyzed the GFP signals following Dox treatment. Confocal microscopy of the Tet-Off-GFP-*MoVPS35* and Tet-Off-GFP-*MoVMA1* transformants showed clear GFP fluorescence signals, which diminished after treatment with Dox (**Figures 2E, F**). Quantification of fluorescence intensity in the areas with and without hyphae confirmed a significant decrease in GFP signals after the addition of Dox, consistent with the background fluorescence levels (**Figures 2G, H**). Furthermore, Dox treatment did not affect the localization or fluorescence intensity of multiple marker proteins in the wild-type strain (**Supplementary Figure S3**). These results indicate that the Tet-Off-GFP transformants provide a straightforward and effective method for monitoring target protein levels under Tet-Off regulation.

### The Tet-Off system can be used to determine the subcellular localization of proteins in *M. oryzae*

The Tet-Off system serves as a highly effective *in situ* tool for observing the subcellular localization of target proteins through N-terminal GFP insertion. The vector map used is shown in **Supplementary Figure S4**. Using a laser scanning confocal microscope, we examined the localization of Tet-Off-GFP transformants. Colocalization with the vacuole membrane marker MoRab7 in vegetative hyphae revealed that GFP-MoVps35 was primarily localized in a punctate pattern around the vacuole, consistent with previous studies (**Figure 2I**) (Chen et al., 2024). GFP-MoVma1 localized to the cytoplasm and on the vacuole membrane (**Figure 2J**). Subcellular localization was also observed in spores, appressoria, and infective hyphae (**Supplementary Figure S5**). These results provide further insight into the spatial dynamics of these essential proteins, enhancing our understanding of their roles in fungal cellular processes.

### Analyzing the growth and pathogenicity of the Tet-Off strains

**Figure 3:**
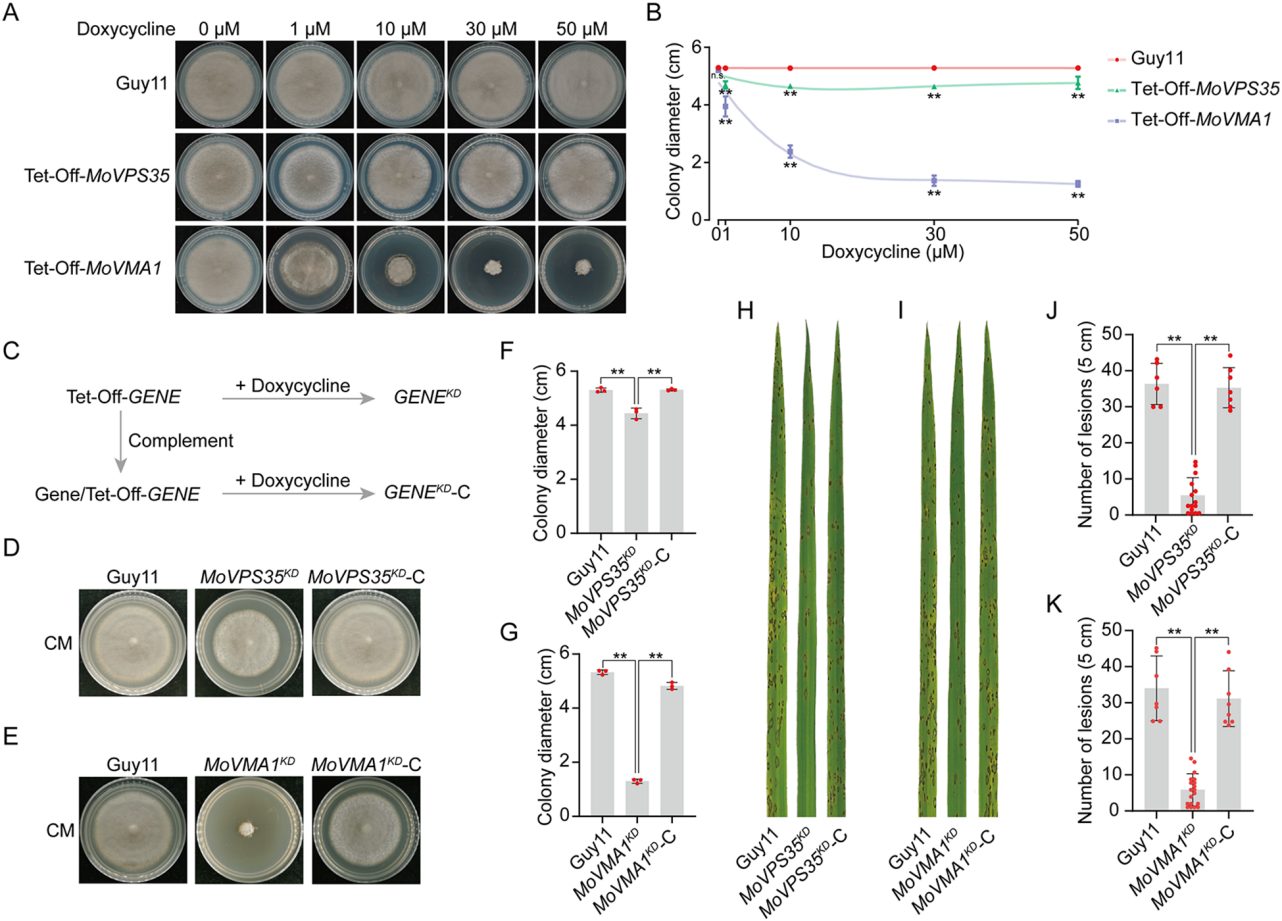
Phenotypic analyses of *MoVPS35*- and *MoVMA1*-Tet-Off mutants. **(A)** Vegetative growth of *MoVPS35-* and *MoVMA1-*Tet-Off mutants on CM media containing different concentrations of Doxycycline. **(B)** Colony diameters of *MoVPS35-* and *MoVMA1-*Tet-Off mutants on CM media supplemented with different concentrations of Doxycycline, along with their non-linear regression curves (Student’s *t*-test: **, *P* < 0.01). **(C)** Nomenclature of the Tet-Off mutants upon doxycycline addition. **(D)** Vegetative growth of *MoVPS35^KD^* mutant on CM medium. **(E)** Vegetative growth of *MoVMA1^KD^* mutant on CM medium. **(F)** Colony diameters of the wild-type (Guy11), *MoVPS35^KD^* and *MoVPS35^KD^*-C strains on CM media supplemented with Doxycycline (Student’s *t*-test: **, *P* < 0.01). **(G)** Colony diameters of the wild-type (Guy11), *MoVMA1^KD^* and *MoVMA1^KD^*-C strains on CM media supplemented with Doxycycline (Student’s *t*-test: **, *P* < 0.01). **(H)** Pathogenicity of the wild-type (Guy11), *MoVPS35^KD^* and *MoVPS35^KD^*-C strains on rice leaves at 5 days post infection (dpi). **(I)** Pathogenicity of the wild-type (Guy11), *MoVMA1^KD^* and *MoVMA1^KD^*-C strains on rice leaves at 5 dpi. **(J)** Number of lesions on 5 cm from the leaf tip of rice leaves 5 days after infection with wild-type (Guy11), *MoVPS35^KD^* and *MoVPS35^KD^*-C (Student’s *t*-test: **P < 0.01). **(K)** Number of lesions on 5 cm from the leaf tip of rice leaves 5 days after infection with wild-type (Guy11), *MoVMA1^KD^* and *MoVMA1^KD^*-C (Student’s *t*-test: **P < 0.01).

To investigate whether the Tet-Off system functions effectively, we measured and compared the colony growth as well as the pathogenicity of the mutants in relation to the wild-type strain. When cultured on CM media containing varying concentrations of doxycycline (Dox), we observed that the growth of the wild-type strain Guy11 remained unaffected (**Figures 3A, B**). In contrast, the growth of the Tet-Off-*MoVPS35* mutant was slightly inhibited in the presence of Dox, similar to its growth phenotype presented in a previous knockout experiment (Zheng et al., 2015). For the Tet-Off-*MoVMA1* strain, the growth inhibition was more pronounced, particularly with increasing concentrations of Dox. Notably, when Dox concentration reached 30 μM, the effect on colony size was relatively strong and stable, indicating a saturation point for growth inhibition (**Figures 3A, B**). Furthermore, on Dox-supplemented plates, the growth of the Tet-Off-*MoVMA1* strain was inhibited for up to 14 days. The strain then exhibited a slightly faster growth rate between days 14 and 21, but still slower than the wild-type (**Supplementary Figure S6**).

Subsequently, the Dox-induced knockdown state of the Tet-Off transformants will be designated as *GENE^KD^*, the complemented strains under Dox treatment as *GENE^KD^*-C while the wild-type strain remains Guy11 (**Figure 3C**). After five days of culture on CM media, the *MoVPS35^KD^* mutant exhibited slight growth inhibition, while the corresponding complemented strain (*MoVPS35^KD^*-C) showed normal growth (**Figures 3D, F**). In contrast, knockdown of *MoVMA1* severely inhibited the fungal mycelial growth, with the complemented strain showing no significant changes in vegetative growth compared to the wild-type strain under Dox treatment (**Figures 3E, G**). For pathogenicity assays, the knockdown of *MoVPS35* resulted in a significantly reduced virulence, while complementation of the gene resulted in restoration of pathogenicity to levels comparable to the wild-type Guy11 (**Figures 3H, J**). Similarly, the tetracycline-based knockdown of *MoVMA1* also led to significantly reduced virulence, and the complemented strain exhibited restored pathogenicity to levels comparable to the wild-type Guy11 (**Figures 3I, K**). Collectively, these results validate the productiveness of the Tet-Off system in studying essential and pathogenicity-related genes in *M. oryzae*, offering a powerful tool for effective functional genomics research in plant pathogenic fungi.

### The Tet-Off system effectively silenced target of rapamycin in the rice blast fungus

Target of rapamycin (*TOR*) is considered indispensable in both *Fusarium graminearum* and *M. oryzae* (Yu et al., 2014; Marroquin-Guzman and Wilson, 2015). Some previous studies used rapamycin to inhibit *TOR* activity in the rice blast fungus (Marroquin-Guzman et al., 2017; Zhang et al., 2021; Zhu et al., 2021; Li et al., 2023). However, researchers have also attempted to silence *MoTOR* using RNA interference (RNAi). Unfortunately, traditional RNAi methods failed to effectively knock down *MoTOR* under normal culture conditions, with silencing only observed in minimal medium supplemented with sodium acetate (NaAc) (He et al., 2018). This specialized environment significantly affects the growth and behavior of *M. oryzae*, introducing variables that can complicate the interpretation of experimental results.

To overcome this challenge, we constructed a Tet-Off vector targeting *MoTOR* and successfully generated *MoTOR*-Tet-Off transformants. Upon treatment with Dox, we observed a significant reduction in colony size compared to non-induced conditions (**Supplementary Figures S1F, G**). Additionally, we measured the relative expression levels of *MoTOR* in Guy11 and *MoTOR*-Tet-Off transformants upon Dox treatment. The results showed that the Tet-Off system effectively knocked down *MoTOR* under normal culture conditions (**Supplementary Figure S2C**). These findings demonstrate that the Tet-Off system is highly effective for studying essential genes, such as *MoTOR* and *MoVMA1*, that appear challenging to target using RNA interference. It also provides a powerful tool for advancing research into the function of critical genes in *M. oryzae* as well as other model fungal systems or pathogens.

## Discussion

In this study, we successfully applied the Tet-Off system to regulate gene expression in *M. oryzae*, achieving conditional knockdown of target genes. The Tet-Off system offers advantages such as reversibility, specificity and high efficiency, enabling more stable and precise control of gene expression, making it particularly effective for studying the functions of essential genes. Compared to traditional techniques like homologous recombination knockout, which require screening a large number of transformants to identify positive clones, the Tet-Off system significantly alleviates these issues and provides a more reliable approach. Another distinct advantage of the Tet-Off system is its ability to selectively suppress gene expression during specific developmental stages of *M. oryzae*, a capability unattainable with other methods (Chen et al., 2023, 2024). Furthermore, this system holds potential for integration with other genetic tools. For instance, combining it with CRISPR/Cas9 system could enhance transformation efficiency (Li et al., 2025). Given that Cas9 protein is generally considered toxic to *M. oryzae*, strategies such as regulating its transcription using Tet-Off system and subsequently removing the Cas9 sequence via the Cre/loxP system after gene knockout could be employed (Foster et al., 2018). This would enable more advanced studies of gene function and drug target identification.

Despite its advantages, the Tet-Off system has certain limitations. The system relies on tetracycline or doxycycline (Dox) as inducers, allowing precise control of gene expression by adding or removing Dox. Although Dox has a longer half-life compared to tetracycline, regular supplementation is still necessary for long-term experiments (Cunha et al., 1982). However, our results showed that Dox could inhibit the growth of rice blast fungus for up to 14 days and even up to 21 days. This may be related to the concentration used, as the degradation of Dox at this concentration had no significant effect on the rice blast experiment during the experimental period (**Supplementary Figure S6**). Additionally, while Dox exhibits excellent membrane permeability in most eukaryotic organisms and generally does not significantly affect the host, certain considerations must be taken into account. The concentration of the Dox and the sensitivity of the fungus to it may impact the reproducibility of experimental results (**Supplementary Figure S7**). Future experimental designs should account for these factors to avoid unwanted outcomes related to Dox use.

Another limitation is that we have not yet tested the Tet-Off system across a range of environmental conditions. Factors such as nutrient availability, temperature and other environmental variables could influence the stability and efficiency of gene induction. Comprehensive testing in diverse environments will be necessary to fully understand the system’s robustness and adaptability.

In the construction of the Tet-Off system, production of tTA is driven by the promoter of the target gene, which then binds to the downstream *tetO* sequence to activate the adjacent minimal promoter *mfa1*, thereby driving the expression of the target gene. This setup, however, can lead to overexpression of the target gene. While the overexpression of many genes may not significantly impact the host, it may still alter the normal cellular state in certain cases. For such instances, careful induction with a low dose of Dox can be used to fine-tune gene expression, achieving a balance that minimizes overexpression while maintaining the target gene’s native function.

In addition to the successful application of the Tet-Off system in *M. oryzae*, attention should also be paid to the Tet-ON system. The Tet-ON system works in the opposite way to the Tet-Off system: in the absence of Dox, the expression of the target gene remains inactive, and gene expression only occurs after Dox induction (Das et al., 2016). This property makes the Tet-ON system particularly effective for transient gene expression, as it eliminates the need for continuous administration of Dox to suppress gene expression and thus avoids the complications associated with the removal of Dox and its biological half-life. In the Tet-ON system, some amino acids in tTA are altered to produce rtTA, which binds to *tetO* only in the presence of Dox (Gossen et al., 1995). Although this mutation reverses the control mechanism, it significantly reduces the sensitivity of the system to Dox, so that higher concentrations of the inducer are required to activate gene expression. This reduced sensitivity can also lead to a delay in gene expression (Baron and Bujard, 2000). We have attempted to use the Tet-ON system in *M. oryzae*, but have not yet obtained conclusive results. It is unclear whether the failure is due to technical challenges or inherent limitations of the system. Further investigation and optimization are required to determine the viability of the Tet-ON system in this fungal model.

In summary, this study demonstrates that the Tet-Off system is a simple, reproducible, and rapid method for transcriptional regulation of genes in *M. oryzae*. It exhibits superior gene regulatory function for both knockout-compatible genes (e.g., *MoVPS35*) and essential genes (e.g., *MoVMA1*, *MoTOR*). Finally, we encourage researchers working on functional genomics in *M. oryzae* to adopt, develop and optimize this system. This will accelerate research into essential genes and ultimately contribute to effective control of the rice blast fungus.

## Data Availability

The datasets presented in this study can be found in online repositories. The names of the repository/repositories and accession number(s) can be found in the article/**Supplementary Material**.
