# Supplementary material for: Harnessing the Tet-Off system for effective gene silencing in Magnaporthe oryzae
Source: Front Plant Sci. 2025 Sep 12;16:1641730. doi: 10.3389/fpls.2025.1641730 (PMC12463844; doi:10.3389/fpls.2025.1641730)
Supplement: Supplementary file 1 [file DataSheet1.docx]

**Supplementary data**


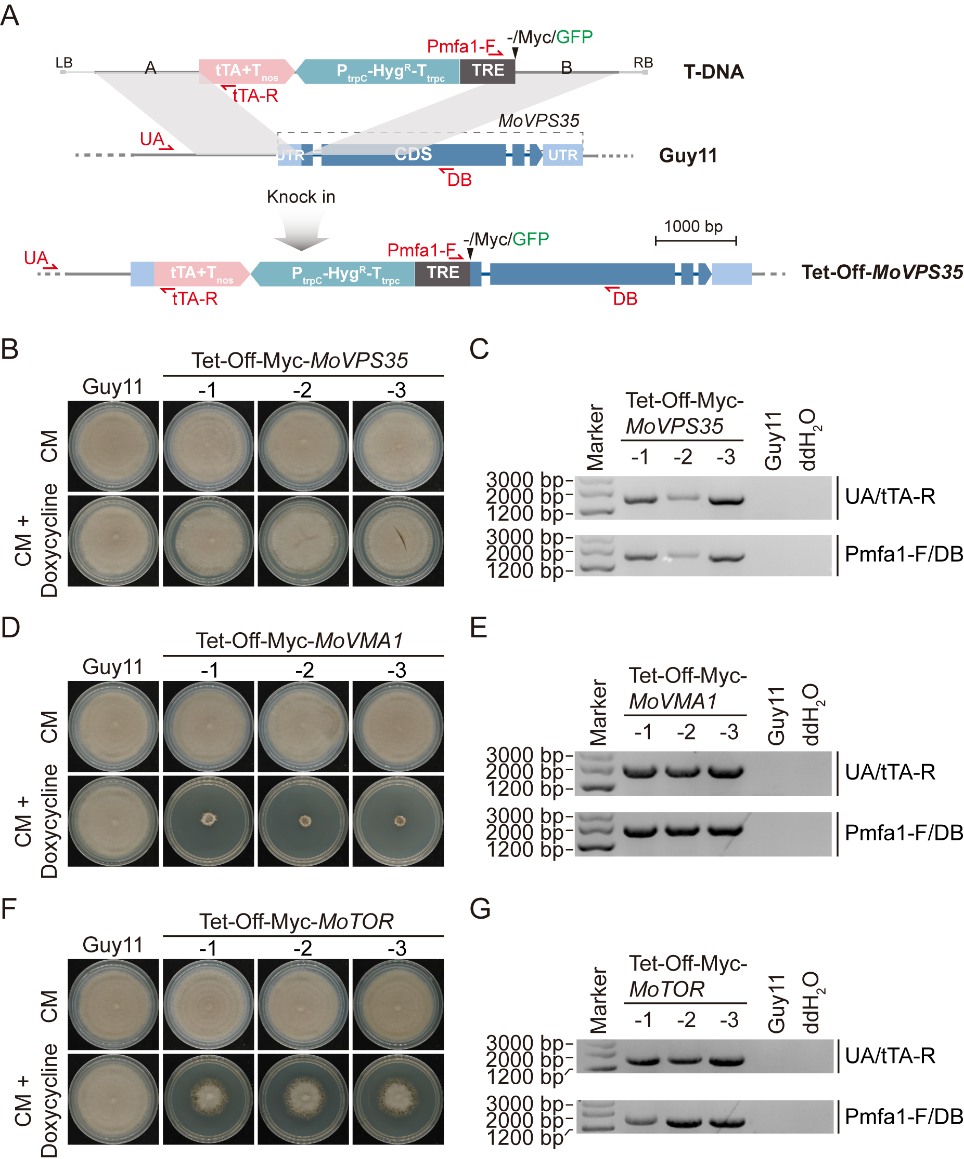


Figure S1: Validation of the generation of Tet-Off transformants in *M. oryzae*. (A) Schematic diagram of primers used to verify the generation of Tet-Off transformants in *M. oryzae*. (B) Vegetative growth of *MoVPS35*-Tet-Off strain on CM medium and CM+Dox medium. (C) Agarose gel electrophoresis of PCR-verified *MoVPS35*-Tet-Off transformants in *M. oryzae*. (D) Vegetative growth of *MoVMA1*-Tet-Off strain on CM medium and CM+Dox medium. (E) Agarose gel electrophoresis of PCR-verified *MoVMA1*-Tet-Off transformants in *M. oryzae*. (F) Vegetative growth of *MoTOR*-Tet-Off strain on CM medium and CM+Dox medium. (G) Agarose gel electrophoresis of PCR-verified *MoTOR*-Tet-Off transformants in *M. oryzae*.

**
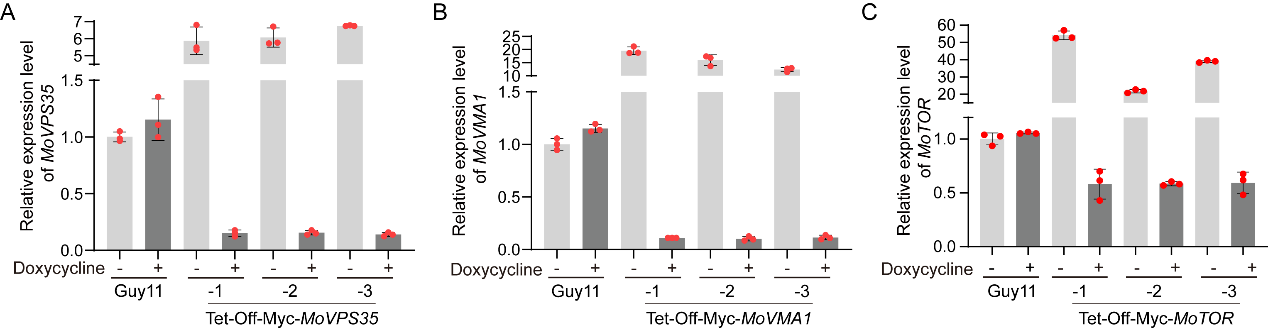
**

Figure S2: Relative transcription levels of targeted genes in the Tet-Off strain with and without Dox addition. (A) RT-qPCR analysis of the relative expression levels of *MoVPS35* in *MoVPS35*-Tet-Off strain. (B) RT-qPCR analysis of the relative expression levels of *MoVMA1* in *MoVMA1*-Tet-Off strain. (C) RT-qPCR analysis of the relative expression levels of *MoTOR* in *MoTOR*-Tet-Off strain.


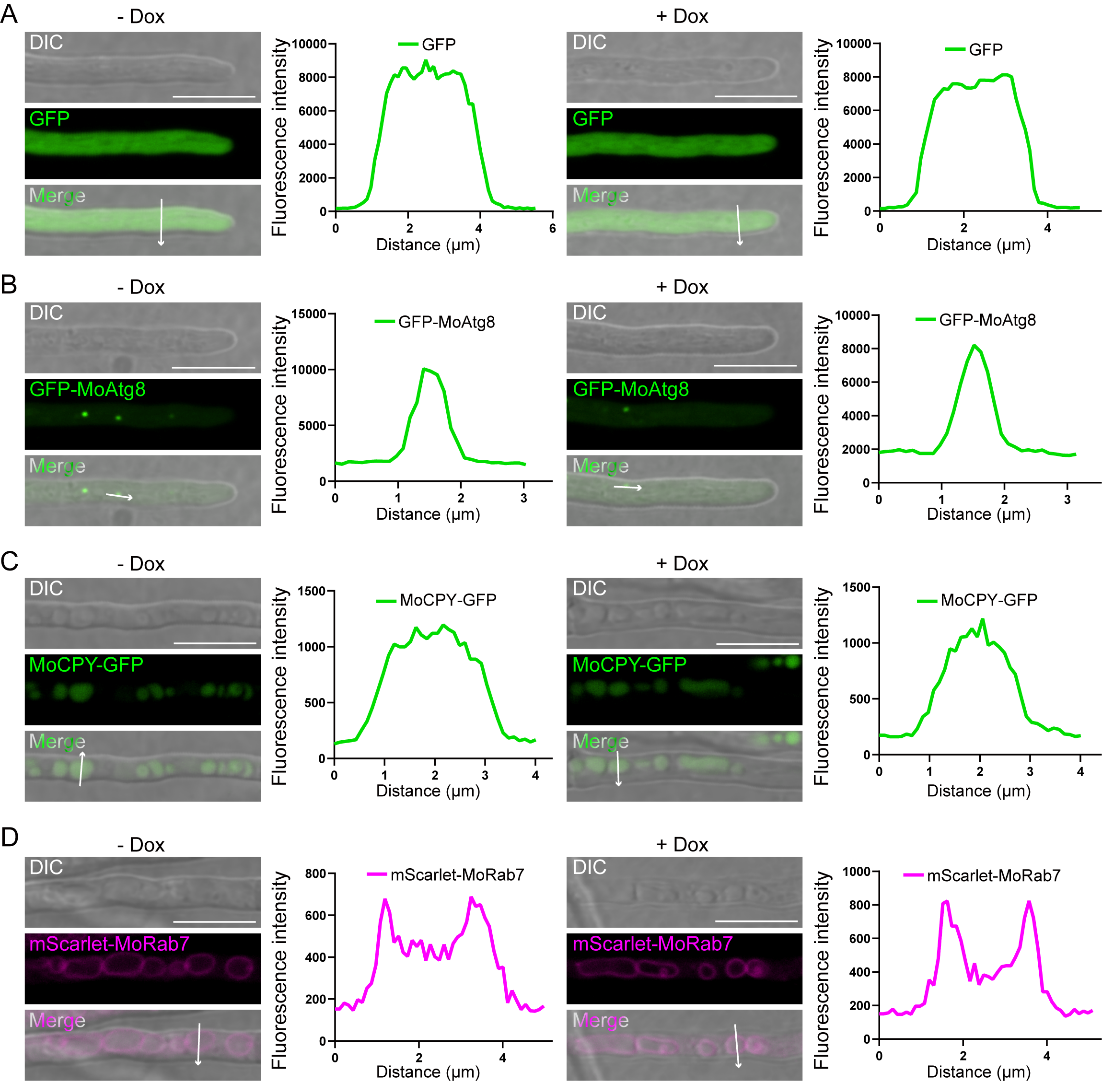


Figure S3: Localization and fluorescence intensity of labeled proteins in *M. oryzae* after Dox treatment. (A) Localization of RP27-GFP after Dox treatment. (B) Localization of GFP-MoAtg8 after Dox treatment. (C) Localization of MoCpy-GFP after Dox treatment. (D) Localization of mScarlet-MoRab7 after Dox treatment.


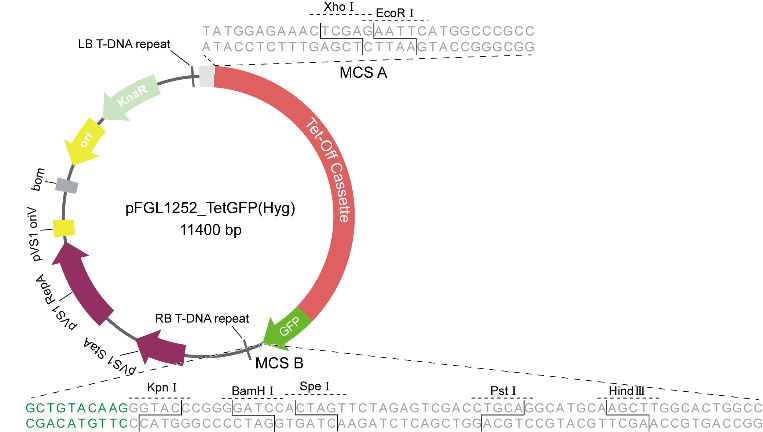


Figure S4: Map and cloning sites of the ATMT vector pFGL1252_TetGFP (Hyg) for Tet-Off-GFP-mediated knock-in.

**
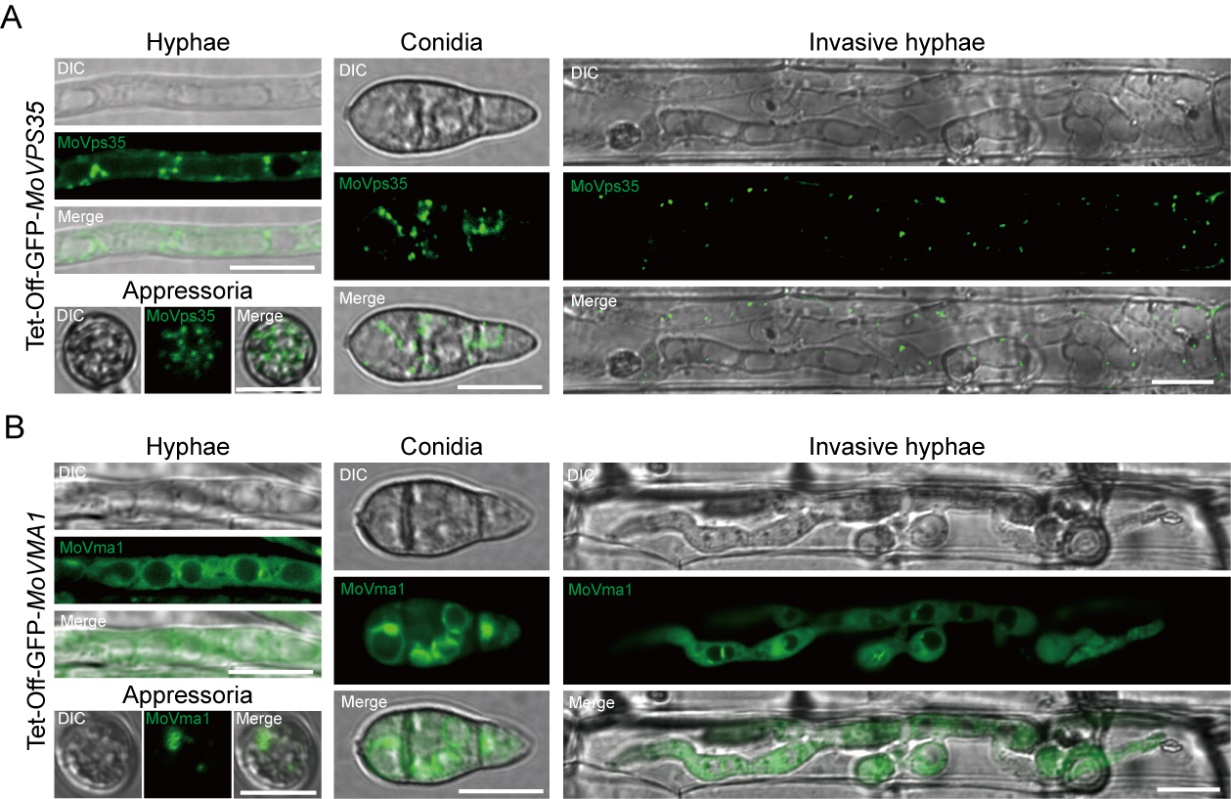
**

Figure S5: Subcellular localization of Tet-Off driven/expressed GFP-tagged proteins at different developmental stages. (A) Localization of GFP-MoVps35 protein at different developmental stages of *M. oryzae*. Bars, 10 μm. (B) Localization of GFP-MoVma1 protein at different developmental stages of *M. oryzae*. Bars, 10 μm.

**
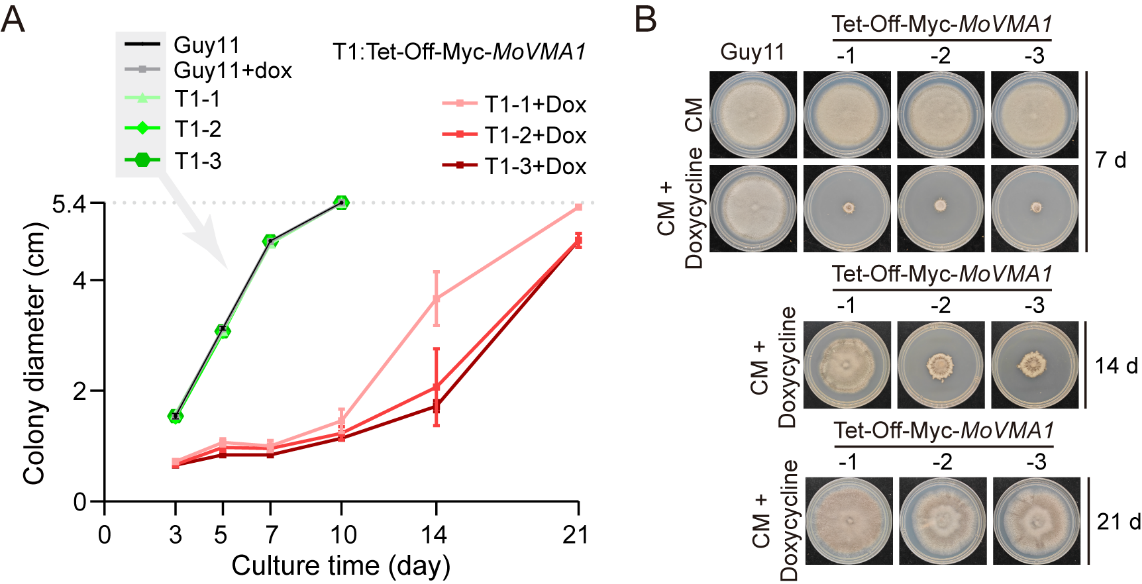
**

Figure S6: Effects on the growth of wild type (Guy11) and *MoVMA1*-Tet-Off strain on CM media with and without doxycycline at the indicated timepoints. (A) Colony diameter of the wild type (Guy11) and *MoVMA1*-Tet-Off strain on CM media with and without doxycycline at different timepoints. (B) Colony morphology of the wild type (Guy11) and *MoVMA1*-Tet-Off mutant on CM media with and without doxycycline at different timepoints.


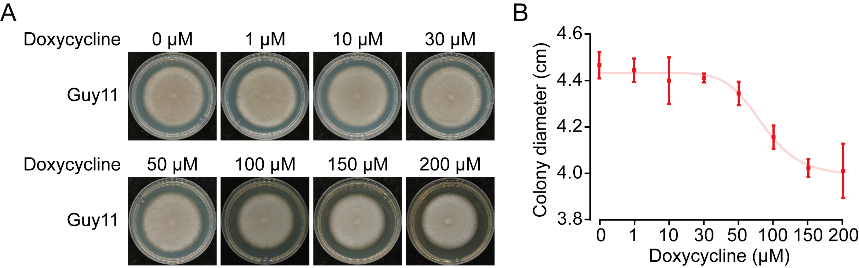


Figure S7: Sensitivity of Guy11 on CM medium with different concentrations of doxycycline. (A) Vegetative growth of Guy11 on CM medium with different concentrations of Doxycycline for 6 days. (B) Colony diameters of Guy11 on CM medium supplemented with different concentrations of doxycycline, along with their non-linear regression curves.


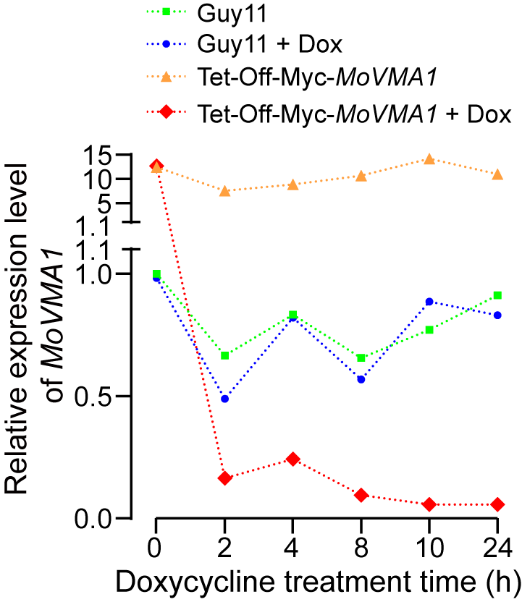


Figure S8: Relative transcript levels of *MoVMA1* in wild type (Guy11) and *MoVMA1*-Tet-Off strain in CM medium with and without doxycycline at different treatment times.

**Table S1**. **Wild-type and transformants strains of fungi used in this study**

| **Strain** | **Genotype description** | **Reference** |
| --- | --- | --- |
| Tetoff-Myc-*MoVPS35* | The Tet-Off cassette and Myc tag were knocked in upstream of the *MoVPS35* ORF in Guy11. | This study |
| Tetoff-GFP-*MoVPS35* | The Tet-Off cassette and GFP tag were knocked in upstream of the *MoVPS35* ORF in Guy11. | Chen et al., 2023 |
| MoVps35-Flag*/*Tetoff-Myc-*MoVPS35* | The MoVps35-Flag_pKNT plasmid was transformed into the Tetoff-Myc-*MoVPS35* strain. | This study |
| Tetoff-Myc-*MoVMA1* | The Tet-Off cassette and Myc tag were knocked in upstream of the *MoVMA1* ORF in Guy11. | This study |
| Tetoff-GFP-*MoVMA1* | The Tet-Off cassette and GFP tag were knocked in upstream of the *MoVMA1* ORF in Guy11. | This study |
| MoVma1-Flag*/*Tetoff-Myc-*MoVMA1* | The MoVma1-Flag_pKNT plasmid was transformed into the Tetoff-Myc-*MoVMA1* strain. | This study |
| Tetoff-Myc-*MoTOR* | The Tet-Off cassette and Myc tag were knocked in upstream of the *MoTOR* ORF in Guy11. | This study |

**Table S2. PCR primers used in this study.**

| **Primers** | **Sequence (5’-3’)** | **Application** |
| --- | --- | --- |
| tTA-R | CGCTTGTTCTTGACGTGCCAG | Primers on the Tet-Off cassette, used for the verification of Tet-Off strains. |
| Pmfa1-F | CCTCAGATCCCGATCAATCCG |  |
| MoVps35-AF | TCACACATTATTATGGAGAAACTCGAGAGTATCTATGGAGGCGGC | For the generation of the *MoVPS35*-Tet-Off vector |
| MoVps35-AR | ATCCAGGCGGGCCATGAATTCGTTGGGAACAAGCTTGCT |  |
| MoVps35-BF(Myc) | TTACTTCTATCAATTGGTACCATGGAGCAAAAGCTCATTTCTGAAGAGGACTTGGCGTCGGTCCCAGCTCCG |  |
| MoVps35-BF(GFP) | GACGAGCTGTACAAGGGTACCATGGCGTCGGTCCCAGCT |  |
| MoVps35-BR | GTCGACTCTAGAACTAGTGGATCCGGCTTCACTGTCAGCTAT |  |
| MoVps35-UA | GAGAGCCTGGTTTCAGAG | Primers on the Guy11, used for the verification of *MoVPS35* Tet-Off strains. |
| MoVps35-DB | CCGGGATAGATGTTGAGC |  |
| MoVma1-AF | ATTATTATGGAGAAACTCGAGAGGGGCTGCTGTTTCCACTC | For the generation of the *MoVMA1*- Tet-Off vector |
| MoVma1-AR | ATCCAGGCGGGCCATGAATTCCTTGATGGAAGGAGCTGGGG |  |
| MoVma1-BF(Myc) | TCAATTGGTACCCGGGGATCCATGGAGCAAAAGCTCATTTCTGAAGAGGACTTGCCTCCGGTAAGCTTCTGA |  |
| MoVma1-BF(GFP) | TACAAGGGTACCCGGGGATCCATGCCTCCGGTAAGCTTCTG |  |
| MoVma1-BR | GCCAAGCTTGCATGCCTGCAGGATGTGTTGGCGATAAGGGT |  |
| MoVma1-UA | TGTCTGCTCCAGTTGTGTCG | Primers on the Guy11, used for the verification of *MoVMA1* Tet-Off strains. |
| MoVma1-DB | TTGGCAGACAGGTAAGCAGG |  |
| MoVps35-Flag F | GGGAACAAAAGCTGGGTACCTGGCAGATGCTTCTCACTCAG | For the generation of the MoVps35-Flag_pKNT vector |
| MoVps35-Flag R | CAGTAACGTTAAGTGGATCCTCACTTGTCATCGTCGTCCTTGTAATCGCTACCACCGCCGCCGCTACCGCCACCACCCTTGGGATCCAACACAAT |  |
| MoVma1-Flag F | GGGAACAAAAGCTGGGTACCCGGGATGAGACCAACAGACAC | For the generation of the MoVma1-Flag_pKNT vector |
| MoVma1-Flag R | CAGTAACGTTAAGTGGATCCTTACTTGTCATCGTCGTCCTTGTAATCGCTACCACCGCCGCCGCTACCGCCACCACCCTCGTCCATGACAGATGC |  |
| MoTor-AF | TCACACATTATTATGGAGAAACTCGAGCGGCACTAAGTAAACCGCCTT | For the generation of the *MoTOR*- Tet-Off vector |
| MoTor-AR | TGACTTATCCAGGCGGGCCATGAATTCTATGGGCAGCCGTGGTGATCC |  |
| MoTor-BF(Myc) | ACTACCTTACTTCTATCAATTGGTACCATGGAGCAAAAGCTCATTTCTGAAGAGGACTTGTCTTCACCAAGCCCAGGCGCA |  |
| MoTor-BR | TGCCTGCAGGTCGACTCTAGAACTAGTCCAGGTATGGTGCCATTTGAC |  |
| β-Tublin qF | CTGCCATCTTCCGTGGAAAG | For the quantification of the related genes expression levels |
| β-Tublin qR | TAGAGCAGAGAGCGGTCTGG |  |
| MoVps35 qF | TCAAATGCTGCTCGACCCTC |  |
| MoVps35 qR | TCGTAGAGGTCGGCAAGATG |  |
| MoVma1 qF | TCTATGTCGGATGCGGCGAG |  |
| MoVma1 qR | CCACGGGCATGTTCGATGTG |  |
| MoTor qF | GCTGTTCAGCGAGACTCCGC |  |
| MoTor qR | CGAATGCGTAGATTCCGCCG |  |

**Table S3. Plasmids used in this study.**

| **Clone** | **Description** | **Reference** |
| --- | --- | --- |
| pFGL1252_Tet-Off(Hyg) | ATMT-mediated transformation vector for the Tetoff strain of *Magnaporthe oryzae* | Vikas Yadav et al., 2019 |
| pFGL1252_TetGFP(Hyg) | ATMT-mediated transformation vector for the Tetoff-GFP strain of *Magnaporthe oryzae* | Vikas Yadav et al., 2019 |
| Myc-*MoVPS35*_pFGL1252_Tet-Off(Hyg) | Fragments targeting *MoVPS35* were fused into the pFGL1252_TetOFF(Hyg) to generate the Tetoff-Myc-*MoVPS35* strain. | This study |
| *MoVPS35*_pFGL1252_TetGFP(Hyg) | Fragments targeting *MoVPS35* were fused into the pFGL1252_TetGFP(Hyg) to generate the Tetoff-GFP-*MoVPS35* strain. | Chen et al.,2023 |
| Myc-*MoVMA1*_pFGL1252_TetOFF(Hyg) | Fragments targeting *MoVMA1* were fused into the pFGL1252_TetOFF(Hyg) to generate the Tetoff-Myc-*MoVMA1* strain. | This study |
| *MoVMA1*_pFGL1252_TetGFP(Hyg) | Fragments targeting *MoVMA1* were fused into the pFGL1252_TetGFP(Hyg) to generate the Tetoff-GFP-*MoVMA1* strain. | This study |
| MoVps35-Flag_pKNT | The MoVps35-Flag fusion was inserted into the pKNT vector for gene complementation. | This study |
| MoVma1-Flag_pKNT | The MoVma1-Flag fusion was inserted into the pKNT vector for gene complementation. | This study |
| Myc-*MoVMA1*_pFGL1252_TetOFF(Hyg) | Fragments targeting *MoTOR* were fused into the pFGL1252_TetOFF(Hyg) to generate the Tetoff-Myc-*MoTOR* strain. | This study |
